# Mating behavior and reproductive morphology predict macroevolution of sex allocation in hermaphroditic flatworms

**DOI:** 10.1186/s12915-022-01234-1

**Published:** 2022-02-07

**Authors:** Jeremias N. Brand, Luke J. Harmon, Lukas Schärer

**Affiliations:** 1 Department of Environmental Sciences, Zoological Institute, University of Basel, Vesalgasse 1, 4051 Basel, Switzerland; 2 Department of Tissue Dynamics and Regeneration, Max Planck Institute for Multidisciplinary Sciences, Am Fassberg 11, 37077 Göttingen, Germany; 3 Department of Biological Sciences, University of Idaho, Life Sciences South 252, 875 Perimeter Dr MS 3051, Moscow, ID USA

**Keywords:** Convergent evolution, Local sperm competition, Sperm competition, Sexual conflict, Sexual selection, Evolution, Comparative morphology, Selfing syndrome, Traumatic insemination

## Abstract

**Background:**

Sex allocation is the distribution of resources to male or female reproduction. In hermaphrodites, this concerns an individual’s resource allocation to, for example, the production of male or female gametes. Macroevolutionary studies across hermaphroditic plants have revealed that the self-pollination rate and the pollination mode are strong predictors of sex allocation. Consequently, we expect similar factors such as the selfing rate and aspects of the reproductive biology, like the mating behaviour and the intensity of postcopulatory sexual selection, to predict sex allocation in hermaphroditic animals. However, comparative work on hermaphroditic animals is limited. Here, we study sex allocation in 120 species of the hermaphroditic free-living flatworm genus *Macrostomum*. We ask how hypodermic insemination, a convergently evolved mating behaviour where sperm are traumatically injected through the partner’s epidermis, affects the evolution of sex allocation. We also test the commonly-made assumption that investment into male and female reproduction should trade-off. Finally, we ask if morphological indicators of the intensity of postcopulatory sexual selection (female genital complexity, male copulatory organ length, and sperm length) can predict sex allocation.

**Results:**

We find that the repeated evolution of hypodermic insemination predicts a more female-biased sex allocation (i.e., a relative shift towards female allocation). Moreover, transcriptome-based estimates of heterozygosity reveal reduced heterozygosity in hypodermically mating species, indicating that this mating behavior is linked to increased selfing or biparental inbreeding. Therefore, hypodermic insemination could represent a selfing syndrome. Furthermore, across the genus, allocation to male and female gametes is negatively related, and larger species have a more female-biased sex allocation. Finally, increased female genital complexity, longer sperm, and a longer male copulatory organ predict a more male-biased sex allocation.

**Conclusions:**

Selfing syndromes have repeatedly originated in plants. Remarkably, this macroevolutionary pattern is replicated in *Macrostomum* flatworms and linked to repeated shifts in reproductive behavior. We also find a trade-off between male and female reproduction, a fundamental assumption of most theories of sex allocation. Beyond that, no theory predicts a more female-biased allocation in larger species, suggesting avenues for future work. Finally, morphological indicators of more intense postcopulatory sexual selection appear to predict more intense sperm competition.

**Supplementary Information:**

The online version contains supplementary material available at 10.1186/s12915-022-01234-1.

## Background

Sex allocation theory predicts how organisms allocate resources to reproduction via their male or female function [1, 2]. Sex allocation has been studied intensively in separate-sexed organisms—generally estimated as the offspring sex ratio [1, 2]—and found to be largely determined by the level of local mate competition (LMC) [3, 4]. LMC occurs in structured populations when only one or a few females lay eggs in a patch (e.g., fig wasps [5, 6]), leading to mate competition between a female’s sons. From the mother’s perspective, making many sons thus wastes reproductive resources that could be reallocated to the production of daughters. Consequently, such mothers produce a female-biased sex ratio to reduce competition between their sons and provide more potential mating partners for them [3, 7].

In contrast, sex allocation in hermaphrodites concerns the allocation of resources by a single individual into its own male or female sex function, either separated in time in sequential hermaphrodites or concurrently in simultaneous hermaphrodites (we focus on the latter, referring to them simply as hermaphrodites). Hermaphroditism is predicted to occur when at least one sex function yields diminishing returns on investment (e.g., the production of sperm or eggs), which then favours resource reallocation to the other sex function, resulting in biased sex allocation [8–10]. It is thought that the male function more often experiences diminishing returns [11]. This results from competition between related sperm, sometimes called local sperm competition (LSC), in analogy to LMC [12]. Specifically, high LSC is expected under monogamy or frequent selfing because both lead to competition between closely related sperm. Sex allocation is thus expected to be strongly female-biased in such organisms [1, 13]. Low population density can also lead to LSC since a reduced encounter probability may decrease the number of (sperm) donors contributing ejaculates to a given (sperm) recipient, and sex allocation is therefore expected to depend on the mating group size [9, 12, 14]. Furthermore, even when mating rates are high, postcopulatory sexual selection mechanisms, such as sperm removal, sperm displacement and/or cryptic female choice, can increase LSC [12, 15–18].

Many hermaphroditic animals and plants exhibit phenotypic plasticity of sex allocation, generally following theoretical predictions [12, 19]. In plants, a more male-biased allocation (i.e., a relative shift towards male allocation) is observed with higher population density, likely because this results in more linear male fitness returns on investment [19–21]. Size-dependent sex allocation is also common and may be linked to the pollination mode. Larger individuals are generally more female-biased in animal pollinated plants but more male-biased in wind pollinated plants [21]. Finally, outcrossing frequency is a strong predictor of sex allocation, with more female-biased allocation under high selfing rates [20]. Similar patterns are found in animals with generally a more male-biased allocation with higher density and/or mating group size (both of which likely correlate with sperm competition intensity) (e.g., [22–25]). Furthermore, there is some evidence of a more female-biased allocation with increasing body size (reviewed in [12]) and with increased selfing [26, 27].

The macroevolution of sex allocation has been studied across numerous taxa in plants (reviewed in [19, 28, 29]). These analyses show an association between the selfing rate and reduced investment into pollen, frequently resulting in convergent changes in flower morphology, which has been termed the selfing syndrome (e.g., [30–32], reviewed in [29]). Sex allocation in plants also depends on the pollination mode, generally showing more male-biased allocation in wind-pollinated plants, potentially because animal pollination limits pollen export [19]. Furthermore, across angiosperm plants, species with larger flowers allocate a larger proportion of total flower biomass to male structures [33].

In contrast to the extensive research in plants, comparative work on sex allocation in hermaphroditic animals is much more limited. A study of six sea bass species found a positive relationship between male allocation and sperm competition intensity, and male allocation was potentially also influenced by spawning frequency and behaviour during gamete release [34]. Moreover, sex allocation was also compared between five goby species. While species always contained some (simultaneous) hermaphrodites with varying allocation to the male function and pure sex females [35, 36], they differed in the presence and frequency of pure sex males, which occurred only in the species with high population density. This suggests that a pure male strategy (i.e., an extremely male-biased allocation) might only occur under intense sperm competition (and thus reduced LSC). Finally, sex allocation was recently investigated in seven *Macrostomum* flatworm species [37]. The results tentatively suggested that the mating behavior and the ability to perform selfing might predict sex allocation (but see below for more detail).

Thus, similar factors predict sex allocation plasticity within species in animals and plants. However, due to the lack of studies, it is unclear if there are general predictors of sex allocation evolution in hermaphroditic animals. A priori, we expect that, like in plants, increased selfing should favor a more female-biased allocation [1, 2, 38]. We also expect–in analogy to the pollination mode–that the reproductive mode, including the mating behaviour, will predict sex allocation. For example, there should be clear differences between internal and external fertilization [12, 39, 40]. Moreover, in internally fertilizing species, we expect factors shaping sperm competition dynamics (e.g., the sperm receiving organ morphology) to predict sex allocation [41]. Describing macroevolutionary predictors of sex allocation in hermaphroditic animals will reveal differences and commonalities between plants and animals and add a missing piece to this successful evolutionary theory [1, 2].

**Fig. 1:**
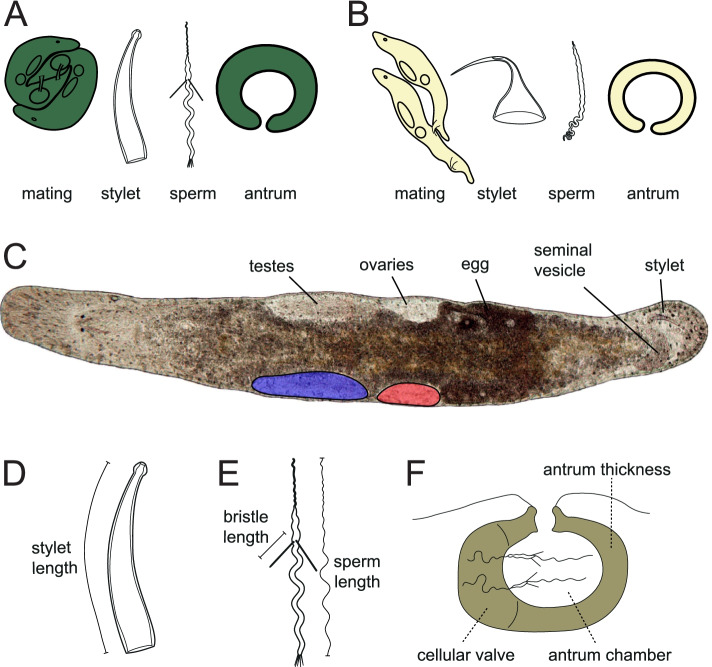
Stylized representation of the mating syndromes, details on the general *Macrostomum* morphology, and the specific in vivo measurements used in the analyses. Most species can be assigned to either the reciprocal mating syndrome (**A**), with reciprocal mating, a (usually blunt) non-invasive stylet, sperm with lateral bristles and a thickened antrum epithelium, or they show the hypodermic mating syndrome (**B**), with hypodermic insemination, a needle-like stylet, simple sperm and a thin antrum epithelium. Note that assignments follow the inferred mating syndrome [43], based on morphological data and observations of the location of received sperm. We use colour throughout to represent species assignment to the inferred mating syndromes: hypodermic (yellow, *N*=40), intermediate (light green, *N*=2), reciprocal (green, *N*=69), and unclear (gray, *N*=9). **C** In vivo image of *M. lignano* with visible internal organs and an indicated outline of how the areas of the paired testes (blue) and ovaries (red) were measured (only one testis and ovary are outlined). These area measurements were used to estimate gonad size and gonadal sex allocation (GSA, as testis area/(testis area + ovary area)). Below are drawings of additional reproductive morphology traits, namely a stylet (**D**), a sperm with sperm bristles (**E**), and an antrum with received sperm (**F**), with representations of the measurements taken or the structures scored, which may represent non-gonadal components of male and female allocation and serve as indicators of the intensity of postcopulatory sexual selection (“morphological indicators”). Linear quantitative measurements were taken as indicated in **D** and **E**. Such quantitative measures were not possible for the antrum (**F**), and we thus scored the indicated structures on an ordinal scale and summed these for an overall measure of antrum complexity (high values indicate a more complex antrum). For details see the “Methods” section.

Here, we search for macroevolutionary predictors of sex allocation across the free-living flatworm genus *Macrostomum*. The worms in this species-rich group of hermaphrodites are microscopic and transparent, allowing in vivo measurements of reproductive trait morphology, and testis and ovary size. From the gonad sizes, we can then calculate gonadal sex allocation (GSA) as the ratio of testis size over total gonad size [42] (Fig. 1C). Moreover, we can assign most *Macrostomum* species to one of two contrasting mating syndromes [43, 44]. These represent convergent combinations of morphological and behavioral characters that indicate different mating behaviours, namely reciprocal copulation or hypodermic insemination (Fig. 1). Additionally, the species show extensive variation in several fascinating reproductive morphologies (Fig. 1D–F) that may serve as indicators of the intensity of postcopulatory sexual selection (“morphological indicators”), including the complexity of the (female) antrum (the female genitalia), the size and shape of the stylet (the male intromittent organ), and different aspects of the sperm design [43–45]. These morphological indicators likely also constitute non-gonadal components of male and female sex allocation (see the “Methods” section).

In species with the reciprocal mating syndrome [43, 44], the donors reciprocally insert their stylet into the recipient’s antrum, which often shows a thickened epithelium (Fig. 1A) and stores the received sperm [46] (Fig. 1F). Furthermore, in many reciprocally mating species, one can observe the so-called postcopulatory suck behavior, where recipients put their mouth on their own female genital opening, probably to remove received ejaculate [45, 47–49]. Such ejaculate removal could allow the recipient to exert cryptic female choice and/or represent a female resistance trait in sexual conflict over the fate of the ejaculate [45]. The sexual conflict interpretation is supported by the presence of stiff lateral sperm bristles, a possible male persistence trait (Fig. 1A, E), which is hypothesized to anchor sperm inside the antrum during the suck behaviour and to thereby defend against the recipient’s sperm removal attempts [45]. Moreover, several seminal fluid proteins identified in the main model species, *Macrostomum lignano*, reduce the recipient’s propensity to perform the suck behaviour [48], further supporting this sexual conflict view. In contrast, species with the hypodermic mating syndrome [43, 44] mate via hypodermic insemination (HI), during which donors traumatically inject sperm into the recipient’s tissues using a needle-like stylet (Fig. 1B). Comparative analyses indicate that HI has convergently evolved at least nine times in the genus [43, 44] and that its emergence coincides with convergent changes in sperm design and both female and male genital morphology. Specifically, species with HI have a simplified antrum, shorter and sharper stylets, and shorter and simpler sperm with either absent or drastically reduced bristles (Fig. 1B) [43, 44].

HI probably affects the intensity and mode of sperm competition and thus the optimal sex allocation, but the direction of the effect is currently unclear [44, 50]. On the one hand, HI could lead to increased male investment. In reciprocally mating species, donated sperm is stored inside the antrum, where it can be exposed to the recipient’s suck behavior and sperm displacement by competing donors, both of which likely increase the realized level of LSC [15, 17, 18]. In contrast, in species with HI, the donor circumvents the antrum, thus possibly reducing the recipient’s ability to control the fate of the received ejaculate. Furthermore, already stored sperm is likely inaccessible to competing donors, hindering sperm removal or displacement. Therefore, under HI, sperm competition could be more fair-raffle like [51], which would reduce LSC and thus favor an increased male allocation [44, 50]. The fact that species with HI have shorter sperm than reciprocally mating species supports this prediction [43]. This is because sperm length and number likely trade-off [52–55], suggesting that under HI, selection favors more numerous but also shorter sperm. On the other hand, HI could lead to decreased male allocation because it facilitates selfing [26, 37, 56–58], leading to a drastic increase in LSC [1, 13]. The hypodermically mating species, *Macrostomum hystrix*, is thought to perform selfing by hypodermically inseminating into its own anterior body region [57]. Several other hypodermically mating species also self [23], but how fertilization is finally achieved is currently unknown, and not all species have been examined. Furthermore, at least one reciprocally mating species, *Macrostomum mirumnovem*, can also self [23], so while HI may facilitate selfing [26, 57, 58], it is not required.

As briefly mentioned above, a previous study investigated GSA evolution in seven *Macrostomum* species [37]. While not conclusive, the data suggested that the inferred mating syndrome and the ability to self might predict sex allocation. Two of the three hypodermically mating species and three of the four selfing species had the most ovary-biased sex allocation. However, the abovementioned hypodermically mating and facultatively selfing *M. hystrix* had the most testis-biased sex allocation [37]. Therefore, the general effect of HI on the evolution of sex allocation remains unclear.

Here we use a recent and robust phylogenetic framework [59] to study the macroevolution of sex allocation across 120 *Macrostomum* species. First, we ask if the mating syndrome predicts GSA. Second, we estimate heterozygosity as a proxy for the degree of inbreeding/selfing to determine how it relates to the mating syndrome and if it predicts GSA. Third, we ask if changes in GSA capture reallocation between the sex functions. Finally, we elucidate if the abovementioned morphological indicators of the intensity of postcopulatory sexual selection can predict GSA.

## Results

### Sex allocation evolution

**Fig. 2:**
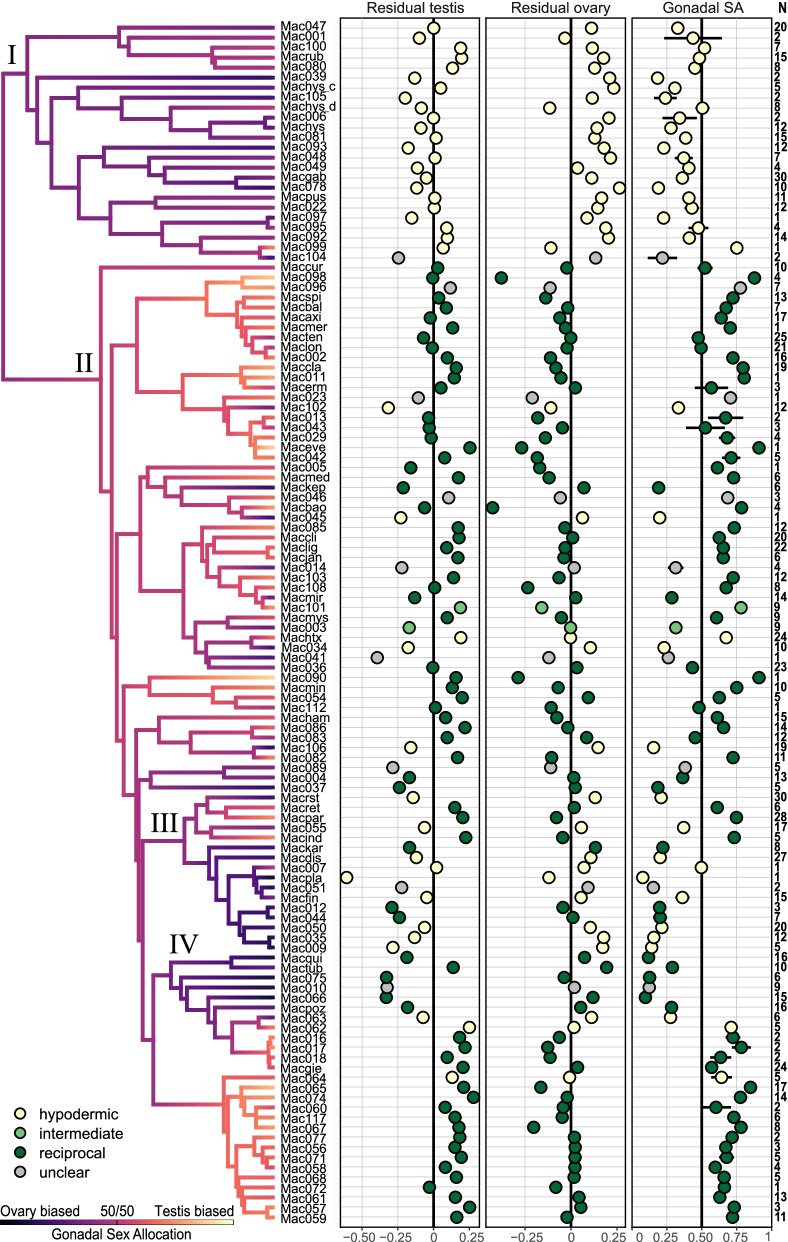
Evolution of gonadal sex allocation (GSA) across 120 species of *Macrostomum*. Colors along the branches show results of an ancestral state reconstruction for GSA. Some clades are marked with Roman numerals (see the “Results” section), and species names are abbreviated (three letters for the genus, and three letters or a three-digit number, respectively, for named species and new species; see Additional file 1: Table S2 for full names). The three panels show, from left to right, residual testis size, residual ovary size, and GSA (i.e., testis area/(testis area + ovary area)), with dots representing species means and whiskers representing standard errors (no SEs are given for the former two since the residuals were calculated across all species using species means). Dot color indicates the inferred mating syndrome that the species are assigned to hypodermic (yellow), intermediate (light green), reciprocal (green), and unclear (gray). The last column (*N*) gives the number of specimens with a GSA estimate. Note that no SEs are drawn for species with one sample only (14 of 120) and in many cases the SEs are small and thus do not extend beyond the symbols

We recently collated a morphological and phylogenetic dataset including 145 *Macrostomum* species from across the globe [43]. For the present study, we estimated the gonadal sex allocation (GSA; Fig. 1C) for 120 of these species (1092 specimens, Additional file 1: Tables S1-S3). While for 26 species, we measured only a few specimens (*N*=1: 14 species, *N*=2: 12 species), we had larger sample sizes for the remaining species (*N*=3–4: 13 species, *N*=5–10: 37 species, *N*>10: 44 species). Furthermore, standard errors of GSA estimates were generally low (Fig. 2), suggesting single specimens represent useful estimates of species means. The arithmetic mean GSA across all species was 0.50 (median 0.52), but species varied considerably, ranging from strongly ovary-biased (0.08) to strongly testis-biased (0.91, Fig. 2). Note that theory predicts absolute allocation should not exceed 0.5. However, GSA measures represent relative rather than absolute allocation (see the “Methods and discussion”). Closely related species tended to have a similar GSA, resulting in non-zero estimates for the indicators of phylogenetic signal lambda (*λ*=0.64) and Bloomberg’s *K* (*K*=0.33), but they covaried less than expected under a Brownian motion model (where *λ* and *K* would equal 1). We found qualitatively similar patterns for residual testis size (*λ*=0.47, *K*=0.23), residual ovary size (*λ*=0.55, *K*=0.47), and body size (*λ*=0.80, *K*=0.39), with all reported values being significantly different from both 0 and 1 (all *P* ≤ 0.01).

Ancestral state reconstruction suggested that the large clade containing only species assigned to the hypodermic mating syndrome (clade I in Fig. 2) had ancestors with a strongly ovary-biased GSA. In clade I, only the single specimen measured for *M.* sp. 99 (Mac099) had a substantially testis-biased GSA (Fig. 2). In contrast, clade II—containing primarily species assigned to the reciprocal mating syndrome—generally had a more testis-biased GSA. Moreover, while two subclades within clade II showed a more ovary-biased GSA (clades III and IV in Fig. 2), also these contained some species with more testis-biased GSA, such as *Macrostomum paradoxum* (Macpar) in clade III, and *Macrostomum gieysztori* (Macgie) and relatives in clade IV.

### Sex allocation is bimodally distributed

**Fig. 3:**
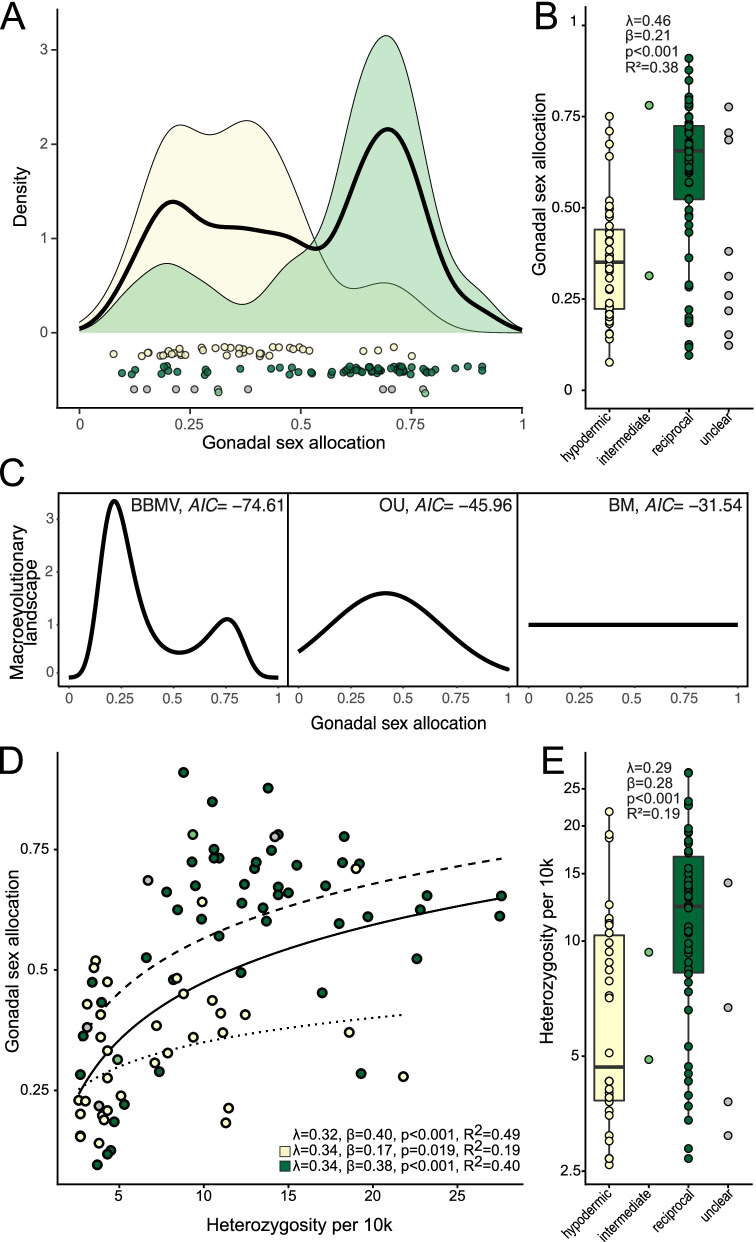
Distribution of gonadal sex allocation (GSA) across all species and links to heterozygosity. **A** The black solid line represents the GSA density across all species, and the colored curves show the densities for the species assigned to the hypodermic (yellow) and reciprocal (green) mating syndrome. Points below show raw data, jittered on the *y*-axis for visibility. **B** Distribution of GSA by inferred mating syndrome. **C** Macroevolutionary landscapes inferred using BBMV. The macroevolutionary landscape represents the normalized evolutionary potential, which determines how fast species evolve towards a trait value (see the “Methods” section). Peaks in the landscape correspond to trait values that species are attracted towards. Given are the three models evaluated with their respective *AIC* values. The full BBMV model was strongly supported. **D** Relationship between heterozygosity and GSA. **E** Distribution of heterozygosity by inferred mating syndrome. Color shows the inferred mating syndrome: hypodermic (yellow), intermediate (light green), reciprocal (green), and unclear (gray). **D** includes the fit and corresponding statistics including all species, as well as separate fits for only the hypodermic and reciprocal mating syndrome, and **B**, **E** include results of comparing the hypodermic and reciprocal mating syndrome (i.e., excluding species assigned as intermediate and unclear). Boxplots show the second and third quartile with whiskers extending up to 1.5 times the interquartile range. *R*^2^ values represent *R*^2^_pred_ of the full PGLS including the phylogeny

GSA was bimodally distributed (Fig. 3A, solid black line), with an ovary-biased peak at 0.21, a testis-biased peak at 0.70, and a local minimum at 0.53. Separating species by inferred mating syndrome shows that the ovary-biased and testis-biased peaks largely correspond to the hypodermic and reciprocal mating syndromes, respectively (Fig. 3A, yellow and green distributions). Almost all species assigned to the hypodermic syndrome had a GSA <0.5, while most species assigned to the reciprocal syndrome had values >0.5.

We explored if peaks in GSA density corresponded to distinct peaks in a macroevolutionary landscape by fitting three evolutionary models to the data using the “Bounded Brownian Motion with potential” approach [60, 61]. Specifically, we assessed whether our data best fit a landscape that is flat (i.e., Brownian motion, BM), has a single peak (i.e., Ornstein-Uhlenbeck, OU), or has two peaks (BBMV, [61]). We found that a two-peak landscape best fit our data (Table 1, Fig. 3C). The preferred model had an ovary-biased peak at 0.22 (Fig. 3C), closely corresponding to the first peak of the measured GSA distribution (Fig. 3A). The model had a second peak at 0.76, which was slightly more testis-biased than the measured peak. Besides determining the shape of the evolutionary landscape, BBMV also estimates how strong the attraction towards peaks is, with larger peaks representing stronger attraction (see the “Methods” and [61]). Interestingly, the ovary-biased peak was three times higher than the testis-biased peak (i.e., 3.34 vs. 1.11), indicating that species quickly evolve towards a low GSA while they do not increase their GSA as rapidly.

**Table 1:** Results from BBMV analysis including gonadal sex allocation of 120 species. For each model, we give the Brownian motion variance (σ^2^), and the values of the parameters of the polynomial terms used to shape the macroevolutionary landscape (*ax*^4^ + *bx*^2^ + *cx*) and the AIC weights. Values in brackets are the bounds of the 95% CI. Note, that parameter b is negative for the BBMV model, which results in a landscape with two peaks

Model	*σ*^2^	*a*	*b*	*c*	*AICw*
BBMV	0.78 (0.55, 1.33)	3.12 (2.73, 3.61)	−4.12 (−4.69, −3.48)	0.68 (0.1, 1.2)	>0.99
OU	0.72 (0.4, 1.04)	-	0.76 (0.37, 1.19)	0.38 (−0.18, 0.73)	<0.01
BM	0.77 (0.28, 1.25)	-	-	-	<0.01

### Hypodermic species have an ovary-biased sex allocation

We tested if the inferred mating syndrome predicted GSA using weighted phylogenetic generalized least squares (PGLS) regression. We found that the hypodermic mating syndrome was associated with a significant reduction in GSA compared to the reciprocal mating syndrome (Fig. 3B, Additional file 1: Table S4). Moreover, this association persisted when we excluded clade I (Additional file 2: Figure S1D, Additional file 1: Table S4), suggesting the result was not primarily driven by this rather uniform clade. Furthermore, we recovered qualitatively similar associations when using other predictors closely associated with the mating syndromes, namely received sperm location, sperm bristle state, and antrum state (Additional file 2: Figure S1A-C, Additional file 1: Table S4).

### Observed heterozygosity predicts sex allocation

Selfing rates are expected to affect GSA, but they have not been estimated for any *Macrostomum* species and obtaining such estimates was outside of the scope of this study. However, regular selfing is expected to reduce heterozygosity, which can be estimated from transcriptome data, giving some indication of genetic diversity across species. Reduced heterozygosity could, however, also be caused by other factors leading to small effective populations size, such as founder effects and biparental inbreeding [62]. We estimated per-site heterozygosity in all specimens with transcriptomes using a superTranscript approach [63] (Additional file 3: Figure S2, Additional file 1: Table S5).

We observed lower heterozygosity in species assigned to the hypodermic mating syndrome (Fig. 3D), consistent with increased selfing in these species leading to reduced effective population size. As expected from these results, we found a significant positive relationship between heterozygosity and GSA across all species (Fig. 3C, Additional file 1: Table S6). The logarithmic fit of our PGLS analysis further revealed that this relationship was non-linear, in that GSA initially increased sharply with heterozygosity but then decelerated. Interestingly, the relationship persisted within both the hypodermic and the reciprocal mating syndrome, with the effect size being smaller in the former (Fig. 3C), suggesting a general link between heterozygosity and GSA.

### Changes in gonadal sex allocation represent reallocation

**Fig. 4:**
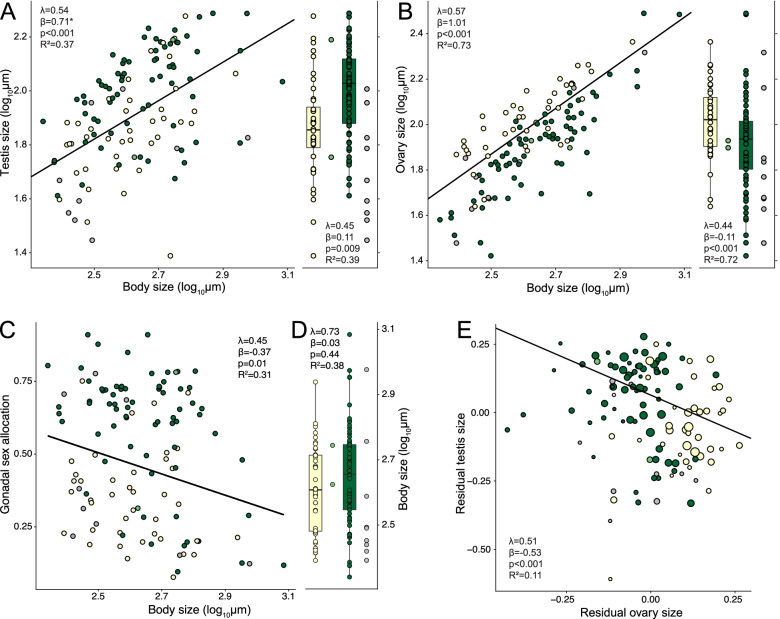
PGLS analysis of body size and gonad size. **A**, **B** Regression of testis size and ovary size on body size (left) and distribution of gonad size by inferred mating syndrome (right). The slope was significantly different from unity for the testis (i.e., negative allometry indicated by an *) but not for the ovary (see Additional file 1: Table S7 for details). **C** Regression of body size on gonadal sex allocation. **D** Distribution of body size by inferred mating syndrome. **E** Regression of residual testis size on residual ovary size. Points are scaled based on sample size to illustrate the weights used for the PGLS model. Color shows the inferred mating syndrome: hypodermic (yellow), intermediate (light green), reciprocal (green), and unclear (gray). Scatterplots include the fit and corresponding statistics for analysis with all species. Boxplots include results of comparing the hypodermic and reciprocal mating syndrome (excluding species assigned as intermediate and unclear) and show the second and third quartile with whiskers extending up to 1.5 times the interquartile range. *R*^2^ values represent *R*^2^_pred_ of the full PGLS including the phylogeny

Because GSA is a ratio, it is affected by changes in testis size, ovary size, or both. Therefore, we performed PGLS regression of both testis and ovary size on body size to investigate whether GSA differences between the syndromes represent reallocation. We either excluded (allometry models) or included (covariate models) the inferred mating syndrome as a predictor. Testis size was positively related to body size, but larger species had proportionally smaller testes (negative allometry, Fig. 4A left, Additional file 1: Table S7A). When correcting for body size, the testis size was significantly larger in reciprocal species than in hypodermic species (Fig. 4A right, Additional file 1: Table S7B). Ovary size scaled isometrically with body size (Fig. 4B left, Additional file 1: Table S7A), and when correcting for body size, hypodermically mating species had larger ovaries (Fig. 4B right, Additional file 1: Table S7B). Therefore, GSA differences between the syndromes resulted from changes in both testis and ovary size. As expected from these results, GSA was negatively related to body size (Fig. 4C, Additional file 1: Table S6), but body size did not differ between the inferred mating syndromes (Fig. 4D, Additional file 1: Table S6). We then also tested for a trade-off between the male and the female gonads using PGLS regression of residual testis size on residual ovary size. Residual testis size was negatively related to residual ovary size (Fig. 4E, Additional file 1: Table S8). Across *Macrostomum*, an increase in ovarian tissue thus coincides with reduced testis tissue.

### Morphological indicators predict sex allocation

**Fig. 5:**
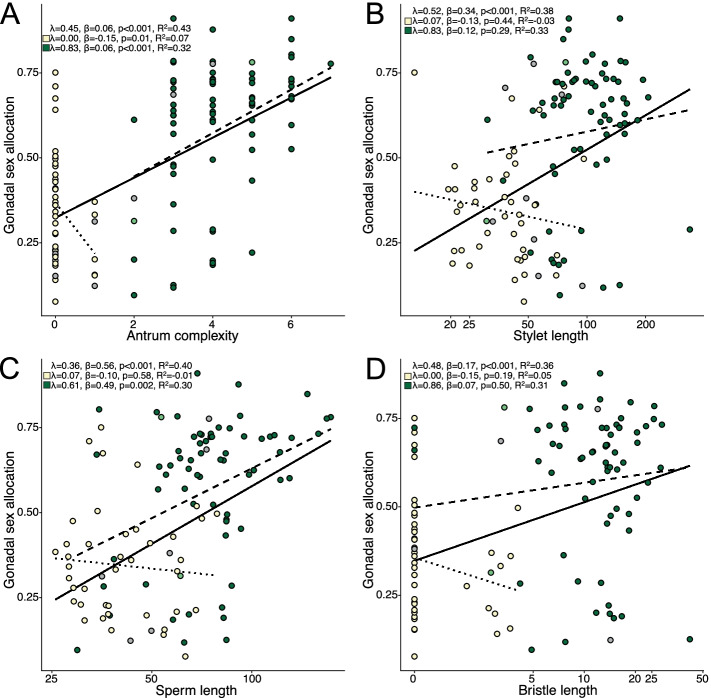
PGLS analysis of four sexual traits against gonadal sex allocation. Each panel (**A**–**D**) includes the fit and corresponding statistics for analysis with all species (solid line, top), only species assigned to the hypodermic mating syndrome (dotted line, middle) or only species assigned to the reciprocal mating syndrome (dashed line, bottom). Color shows the inferred mating syndrome: hypodermic (yellow), intermediate (light green), reciprocal (green), and unclear (gray). Values are plotted on a logarithmic scale in panels **B**–**D** but the axis is labelled with back transformed values. *R*^2^ values represent *R*^2^_pred_ of the full PGLS including the phylogeny. Detailed results are in Additional file 1: Table S6

Next, we explored the relationships between GSA and four sexual traits that are potential morphological indicators of the intensity of postcopulatory sexual selection [43–45], namely antrum complexity, stylet length, sperm length, and sperm bristle length. Across all species, univariate PGLS of sexual traits suggested that GSA was positively related to antrum complexity, stylet length, total sperm length, and bristle length (Fig. 5, Additional file 1: Table S6). A multivariate PGLS, including all sexual traits and body size, suggested a similar pattern, except that bristle length was not significant (Additional file 1: Table S9). Since sperm bristle length was positively related with both stylet length and sperm length (data not shown), its effect was likely masked in the multivariate analysis.

Across species classified as hypodermically mating, only the antrum complexity score was still significant in both the univariate and multivariate analyses (Fig. 5, Additional file 1: Table S6 and Table S9), indicating a negative relationship of GSA with antrum complexity. However, the *R*^2^_pred_ of the univariate model was low (0.07) since only four species had a complexity score >0. In contrast, analysis of species assigned to the reciprocal syndrome broadly recapitulated the findings of the complete dataset. Again, GSA was positively related to antrum complexity and sperm length, in both the univariate and multivariate cases (Fig. 5, Additional file 1: Table S6 and Table S9). Stylet length was not significant in the univariate analysis, but was positively related again in the multivariate analysis, presumably because the multivariate model accounts for body size. To investigate this further, we repeated the stylet length PGLS with body size included as a covariate. Now, stylet length significantly predicted GSA (*λ*=0.6, *β*=0.48, SE=0.13, *t* value=3.8, *p*<0.001), indicating that relative, not absolute, stylet length predicts GSA.

## Discussion

### Sex allocation and hypodermic insemination

Across the genus *Macrostomum*, gonadal sex allocation (GSA) is bimodally distributed. Since the intensity of local sperm competition (LSC) is likely the main driver of GSA evolution, this indicates that most species are exposed to either fairly low or high, but not to intermediate, intensities of LSC. By fitting evolutionary models, we showed that the two modes in the GSA distribution correspond to two adaptive optima on a macroevolutionary landscape, with rapid evolution towards the peak of ovary-biased GSA and slower evolution towards the testis-biased peak. Thus, across the genus GSA primarily becomes more ovary-biased and only rarely becomes more testis-biased. Furthermore, we found that the type of mating behaviour exhibited by the species explained the bimodal distribution of GSA well, with most species with the hypodermic mating syndrome having an ovary-biased GSA, while species with the reciprocal mating syndrome usually have a testis-biased GSA. Across the genus, hypodermic insemination (HI) has evolved multiple times but there is no clear evidence for reversions to reciprocal copulation, suggesting that HI canalizes morphology and could lock species in this mating behavior [43]. The evolutionary model of GSA suggests that HI has a similarly canalizing effect on sex allocation.

Our findings suggest that HI is associated with processes that lead to reduced male allocation [1, 13]. Additionally, they suggest a more gradual change of sex allocation in reciprocally mating species, potentially in response to the observed male-female coevolution in these species [43, 50]. A reduced male allocation in species with HI contradicts the prediction that HI may lead to a more male-biased sex allocation by reducing the potential for sperm displacement and cryptic female choice and thus leading to more fair-raffle sperm competition [44, 50]. Instead, HI could be linked to the ability to perform selfing [37, 56–58], and to perhaps even prefer selfing, with some species showing no evidence of inbreeding depression or delayed selfing [58]. Furthermore, we have established laboratory cultures of several additional species in the hypodermically mating clade (I in Fig. 2) from single individuals, suggesting that selfing could be pervasive in this clade (Brand and Schärer, pers. obs.). Finally, several reciprocally mating species in clade II (Fig. 2) appear to be unable to self (Singh and Schärer, pers. obs.). In agreement with this notion, we observed lower heterozygosity in species assigned to the hypodermic mating syndrome, further supporting the link between HI and selfing.

Naturally, reduced heterozygosity alone is not conclusive evidence of selfing. It simply indicates a smaller effective populations size, which could also result from biparental inbreeding or founder effects [62]. Therefore, hypodermically mating species could also have an ovary-biased GSA because they mostly occur in small fragmented populations. Unfortunately, little is known about the ecology of *Macrostomum*, partially due to the extensive effort required in sample extraction. Our field collections indicate that population densities vary from one to hundreds of animals in 100 ml of sediment/substrate, and we have collected both hypodermic and reciprocally mating species across the density spectrum (Brand and Schärer, pers. obs.). Alternatively, HI could lead to LSC even in dense populations if injected sperm only rarely encounter rival sperm (e.g., if once injected, hypodermic sperm rapidly became non-functional). Unfortunately, no data on the rate of hypodermic sperm transfer or sperm longevity is currently available for any hypodermic species.

#### Does hypodermic insemination constitute a selfing syndrome?

The observed convergent reduction in sex allocation and heterozygosity, combined with replicated changes in sexual traits [43], is reminiscent of the selfing syndrome in plants. The selfing syndrome is defined as an association between the selfing rate and reduced investment into pollen, in combination with convergent changes in flower morphology (e.g., [30–32, 64–66], reviewed in [29]). In hermaphroditic *Biomphalaria* snails, a reduced waiting time (i.e., the time that an isolated individual waits before it resorts to selfing), combined with reduced inbreeding depression, has analogously been called the selfing syndrome [67–69], but few morphological traits have been assessed to evaluate correlated changes. In *Caenorhabditis* nematodes, hermaphroditic selfing lineages have originated a least three times [70] and these origins are associated with replicated behavioral, morphological, and genomic changes [71, 72].

In agreement with our inferred link between selfing and reduced male allocation, some hermaphroditic animals reduce their male allocation under selfing [26, 27], but others show no or inconsistent patterns [68, 73]. However, reduced male allocation could also result from other factors. For example, in the free-living flatworm *Schmidtea polychroa*, populations with sperm-dependent parthenogenesis show reduced male allocation compared to outcrossing populations, presumably due to limited outcrossing opportunities in the former [74, 75]. Assuming the observed reduction in heterozygosity results from selfing, our data suggest that HI constitutes a selfing syndrome. Selfing may thus be a wide-spread phenomenon across different groups of flatworms [76–79] and other hermaphroditic invertebrates [80–82] that have evolved HI. It would further make it likely that the selfing rate can predict sex allocation in hermaphroditic animals generally.

It has been suggested that transitions to selfing are irreversible, leading to lineages suffering an increased risk of extinction and thus representing an evolutionary “dead-end” [83, 84]. However, others have argued that the reproductive isolation and increased dispersion potential resulting from selfing can drive speciation [71]. Our data supports both hypothesis to some extent. On the one hand, we observe several single species with HI at the tips of the phylogeny. On the other hand, we see multiple clades with HI that are old (but note that due to the lack of fossils in these small invertebrates no calibrated phylogeny is available). Particularly, in the hypodermic clade (Fig. 2, clade I), extensive (cryptic) speciation has occurred [59]. This suggests that the hypodermic clade has purged inbreeding depression, which is supported by a loss of waiting time and no obvious inbreeding depression in one member of this clade (*Macrostomum pusillum*, [58]).

#### Broader implications on sex allocation theory

We find a negative relationship between residual testis and ovary size, supporting the trade-off assumption, a central tenet of sex allocation theory [1, 11]. Moreover, we show that our GSA estimates do not simply reflect changes in, for example, relative testis size, but that they instead indicate reallocation and thus changes in sex allocation. Since theory predicts that a male allocation >0.5 should destabilize hermaphroditism [8], our GSA estimates likely represent the relative rather than the absolute ratio of resource allocation. Mature eggs are large in *Macrostomum* (up to ~10% of body size) and yolk provisioning, which largely occurs once the oocytes have left the ovary, likely accounts for a substantial fraction of female allocation [85, 86]. Therefore, our GSA estimate likely omits a larger proportion of female compared to male allocation. We did not include developing or mature eggs in our sex allocation estimate because their presence and size can be temporally variable (e.g., in measures are made just before or after egg laying), while ovary size is relatively constant.

It should be noted that although we find a significant sex allocation trade-off, the variance explained by the model was relatively low (*R*^2^_pred_ = 0.11). Therefore, investment into testes vs ovaries may, at least to some extent, be decoupled. Evidence for the trade-off assumption is equivocal in plants (reviewed in [19, 28]) and rather scant in animals (reviewed in [12]). This is unsurprising because a large between-individual variance in resource budget will often result in a positive covariance between traits, even if an underlying trade-off exists [87–89]. While comparative analyses using species means are potentially better suited to reveal such trade-offs, results can still be affected by variable acquisition ability between species [90], offering a possible explanation for the apparent decoupling between ovaries and testes. Furthermore, resources could also be reallocated to other non-gonadal sex allocation components (see the “Discussion” section below).

Interestingly, we show that testis size exhibits negative allometry, while ovary size scales isometrically. Consequently, we find that larger *Macrostomum* species tend to have a more ovary-biased GSA, revealing a form of size-dependent sex allocation (reviewed in [12]). This between-species effect recapitulates the intraspecific finding in *Macrostomum lignano* [91]. Another form of size-dependent sex allocation is found in angiosperm plants, where species with larger flowers have an increased male allocation to flower biomass, most likely due to male-male competition for pollinators [33]. Similarly, selfing plant species tend to have smaller, more female-biased flowers [29]. However, a species’ mean size does not seem to predict sex allocation (e.g., [92]). Within populations, sex allocation has been predicted to be negatively related to body size [93, 94], but because these are intraspecific models, it is not clear what relationship is expected in between-species comparisons. One explanation for our findings is that antrum size and consequently sperm storage capacity show a negative allometry with body size. Given some simplifying assumptions (e.g., constant mating rate and isometric scaling of sperm size), this would limit how much sperm a worm can donate and lead to faster diminishing returns on investment into sperm production in larger species. To test this hypothesis, it would be necessary to quantify antrum size. However, such measurements are challenging because the antrum is highly transparent and flexible. Furthermore, theoretical work should parameterize our argument to generate general macroevolutionary predictions.

#### Sex allocation and morphological indicators

Across all species and restricted to species mating reciprocally, antrum complexity, stylet length, and sperm length were positively related to GSA. A positive relationship between sperm length and testis size is found in several species (e.g., [95–97], but see [98]) and does not necessarily imply that selection acts both on sperm size and number. Instead, selection for longer sperm could result in slower spermatogenesis, requiring a larger testis to produce an equal number of sperm [96, 99–102].

The morphological indicators may represent components of non-gonadal sex allocation. Integrating them into a more inclusive measure of sex allocation would mean that in species with high GSA, male allocation would be further increased due to the sperm and stylet traits, but at the same time, female allocation would be increased due to greater antrum complexity likely also representing higher allocation. Integrating non-gonadal components would be desirable and could potentially explain the observed decoupling between male and female allocation. However, as mentioned earlier, several other sex allocation components (e.g., yolk and seminal fluid production) would also have to be considered to capture a more complete measure of sex allocation.

All measured morphologies likely represent adaptations to postcopulatory processes, such as cryptic female choice (antrum complexity and sperm design), sperm removal (stylet morphology and sperm design), and control over the mating duration (antrum and stylet morphology) and thus should be good proxies for the intensity of postcopulatory sexual selection [43, 44, 91]. Furthermore, we recently showed covariation between male and female genital morphology in reciprocally copulating species, which points to ongoing coevolution. Because these traits also predict GSA, sperm competition likely increases with the intensity of postcopulatory sexual selection. Interestingly, stylet length, sperm length, and bristle length were not significantly related to GSA in hypodermic species and antrum complexity even showed a weak (and non-significant) negative relationship. Therefore, selection on components relevant to postcopulatory sexual selection may cease or even reverse under HI. Because under HI the stylet and sperm no longer interact with the antrum [45, 57, 58], this makes intuitive sense and further supports the view that male-female coevolution drives the evolution of the morphological indicators in reciprocally mating species.

However, while our data is consistent with this interpretation, selfing could also determine GSA in reciprocally mating species. We observed several reciprocally mating species with low levels of heterozygosity, as expected under selfing. While evidence for selfing in these species is scant, one reciprocally mating species, *M*. *mirumnovem*, can self under laboratory conditions (albeit with reduced fecundity of isolated animals compared to pairs) [23] and it has an ovary-biased GSA (0.29). However, *M*. *mirumnovem* has comparably high heterozygosity (19 sites per 10kb), which seems to contradict the selfing hypothesis. But this could be due to the unusual karyotype and an accompanying whole genome duplication of *M*. *mirumnovem* [103, 104], likely violating the assumptions of our heterozygosity analysis. In summary, we cannot exclude that selfing is also important for GSA evolution in reciprocally mating species, but it is unlikely to be the only predictor.

## Conclusions

The bimodal distribution of sex allocation indicates that hypodermic insemination is associated with reduced GSA, likely because it coincides with increased selfing and/or biparental inbreeding. Therefore, the mating behavior predicts macroevolutionary patterns of sex allocation in *Macrostomum* flatworms. This association between inbreeding and reduced investment into the production of male gametes agrees with finding in plants and with theoretical predictions. We, therefore, provide clear comparative evidence that these predictions can be generalized to hermaphroditic animals. We also find a relative shift towards female allocation in large species, which is currently not predicted by theory, highlighting that more theoretical work is needed. Furthermore, we find that morphological indicators of the intensity of postcopulatory sexual selection are positively related to GSA, suggesting that they are associated with intense sperm competition.

## Methods

### Study species and morphological measurements

Most specimens were collected during multiple field sampling trips across the globe. We have previously given a detailed account of the collected specimens, including image and video documentation [43]. Because these animals are transparent, it is possible to observe the testes, the ovaries, the seminal vesicle, and the male and female genitalia in vivo from animals lightly squeezed dorsoventrally between a coverslip and a microscope slide (Fig. 1C). We have previously collected measurements from these specimens [43], including body size, sperm length, sperm bristle length, and stylet length, by tracing the structures using ImageJ [105] (Fig. 1C–E). Images were calibrated using a stage micrometer. We further categorized the female genitalia on a per species basis to produce a compound complexity score, with increasing values representing more complex genitalia (Fig. 1F). Finally, we previously assigned species to an inferred mating syndrome according to their genital morphology and the location of received sperm [43]. In total, 40 species were assigned to the hypodermic mating syndrome, 69 species to the reciprocal mating syndrome, two species were assigned to an intermediate mating syndrome, and nine species could not be assigned. As discussed in [43], the inferred mating syndrome differs slightly from the mating syndrome originally used in [44], in that the latter included information on behavioral data. Of the unassigned species, three (*M*. sp. 14, *M*. sp. 51 and *M*. sp. 89) had a morphology indicative of hypodermic insemination, but we observed received sperm in the antrum, suggesting they may also represent intermediate states between the syndromes. The other species were not assigned because of contradictory information (*M*. sp. 10) or because we lacked information on reproductive morphology (i.e., no observation of the sperm and/or the antrum). See the supporting information of [43] for a more detailed discussion.

Here we additionally estimated GSA as testis area/(testis area + ovary area) for 120 species (calculated on untransformed gonad area values), with most specimens being documented within a few days after field extraction. We estimated the gonad area by tracing their outline manually using ImageJ (Fig. 1C). Whenever possible, we traced both paired testes and ovaries and used their sums as the datum. When tracing one gonad was impossible due to the animal’s position, but we could confirm it was present, we doubled the value of the measured gonad. When it was unclear if the second gonad was present, we used only the estimate for one gonad as the datum (all described *Macrostomum* species have paired gonads, but individual specimens can sometimes lack some gonads). Gonads were generally traced at 400x magnification (1459 testes, 1366 ovaries), but some at 100x (17 testes, 20 ovaries) or 200x (378 testes, 387 ovaries) when very large, or at 1000x (74 testes, 71 ovaries) when very small. We usually measured gonads at the same magnification within an individual (1001 out of 1102 cases). We only estimated GSA for specimens with active gonads, which we judged by the testes having visible elongating spermatids towards the lumen, and ovaries having visible oocytes, yolk granules at the posterior end of the ovary, and in most cases developing eggs. We also required that specimens had a complete male reproductive system, consisting minimally of a seminal vesicle, a vesicula granulorum, and the stylet. Given the variable body sizes of the species and specimens, we could not standardize the thickness of the squeeze preparation (which is usually done when measuring GSA in studies on laboratory-reared *Macrostomum* [42, 58, 106]), potentially making direct comparisons of testis and ovary area between species problematic. In a small side study, we found that, given their relative nature, GSA estimates were unaffected by squeezing intensity in four species (Brand and Schärer, unpublished). We, therefore, expect GSA estimates to be robust. Note also that the cost of the testis and ovary per unit area may not be equivalent and that the testis area likely approximates allocation towards sperm production better than ovary area does allocation towards egg production, making GSA necessarily a relative measure (see also the “Discussion” section).

Several *Macrostomum* species show considerable phenotypic plasticity in GSA [37], which could be problematic if plasticity were of a similar magnitude to between-species differences. However, a comparative study of sex allocation plasticity in response to mate availability and sperm competition intensity in seven *Macrostomum* species found that interspecific variation in GSA was nearly three times larger than intraspecific variation [37]. Therefore, GSA estimates from field-collected specimens should approximate species properties, and this is further supported by the generally low standard errors of GSA in our data (Fig. 2).

Before statistical analysis, we square root-transformed the body, testis, and ovary area measurements to convert them to a linear size estimate. We then log_10_-transformed these size estimates and all other linear measurements (stylet length, bristle length, sperm length). To compare testis and ovary size directly between species, we then also calculated the residuals of a linear regression of the testis and ovary size on the body size across all species (using geometric species means, testis: *F*_1,118_=36.99, *β* = 0.64, *p* < 0.001, adjusted *R*^2^ = 0.24; ovary: *F*_1,118_=179.8, *β* = 1.04, *p* < 0.001, adjusted *R*^2^ = 0.60). All statistical analyses were performed in *R* (version 3.6.0, [107]).

### Phylogeny

We used a recently generated ultrametric molecular phylogeny of the genus *Macrostomum* that is based on a combination of whole-body transcriptome RNA-Seq (98 species) and Sanger-sequenced *28S rRNA* data (47 species, [59]). While the major phylogenetic groupings were consistent between different inference methods and sequence alignments used, the previous study found some discrepancies in the backbone of the phylogeny [59]. We here present results from a phylogeny, called C-IQ-TREE, made from an alignment containing 385 genes (94,625 amino acids in the trimmed alignment) from the 98 species with a transcriptome, plus a *28S rRNA* fragment including an additional 47 species and calculated with a maximum likelihood approach. To assess whether tree uncertainty influenced our conclusions, we also performed all analyses on two phylogenies that only include species with transcriptomic data available, calculated using a maximum likelihood (H-IQ-TREE) or Bayesian approach (H-ExaBayes). The analyses on these alternate topologies were quantitatively similar and qualitatively identical (this was also the case in the analyses in [43]), and we here only present results from the analysis performed on the more comprehensive C-IQ-TREE phylogeny.

### Estimation of heterozygosity

Heterozygosity is a putative correlate of the selfing rate, because a high selfing rate would be expected to reduce the effective population size (N_e_) and thus lead to a reduction in heterozygosity. In order to obtain estimates of per-site heterozygosity for different species, we generated superTranscripts [63] for each of the transcriptomes assembled for [43], excluding transcriptomes from species without GSA estimates or for which we pooled RNA samples from many specimens, leaving us with 130 transcriptomes across 90 species. We then called haplotypes using the GATK 3.8 RNA variant calling pipeline [108]. Briefly, we mapped the trimmed reads to the superTranscripts using STAR in 2-pass mode, post-processed the alignment using Picard, translated mapping scores using GATK *SplitNCigarReads*, and finally called variants using the GATK *HaplotypeCaller*. We discarded SNP clusters (three or more SNPs within a 35-base sliding window) and low confidence SNPs (FS>30, QD<5), which represents more aggressive filtering than the standard pipeline [108]. We calculated the per-site heterozygosity as the number of SNPs divided by the total number of bases across all superTranscripts. Most assemblies are derived from a single individual, and we thus simply mapped the reads used for the assembly. Eight of the transcriptomes were derived from sequencing data from multiple individuals per species (seven species with three samples and one species with two samples), collected from the same location. In these cases, we called variants for each individual separately by mapping the reads to the species reference transcriptome and averaged estimates for each species. We assessed the repeatability of the estimates by calculating the Intraclass Correlation Coefficient (ICC) for species with more than one sample using the R package *ICC* [109]*.* Repeatability was quite low (*N*=22, *k*=2.78, ICC=0.32, 95%CI=0.04–0.6), but this was mainly due to variation between different sampling sites since the ICC was large when we compared specimens collected from the same site (*N*=13, *k*=2.53, ICC=0.87, 95%CI=0.69–0.96, Additional file 3: Figure S2).

### Evolution of sex allocation

We calculated the empirical density of GSA using the *density* function in R (version 3.5.1) and determined the bandwidth using the method described in [110]. Next, we estimated phylogenetic signal (both lambda, *λ*, as well as Bloomberg’s *K*) in the distribution of GSA using the *phylosig* function from the R package *phytools* (version 0.6, [111]) and conducted likelihood ratio tests to determine if the signal differed from zero (indicating no phylogenetic signal) or 1 (indicating the expected phylogenetic signal under Brownian motion). Next, we calculated ancestral states of GSA using the *contMap* function from *phytools*, which determines maximum-likelihood estimates for all internal nodes using the method of Felsenstein [112].

We used the R package BBMV (version 2.1, [60]) to model GSA evolution across the phylogeny based on the Fokker-Planck-Kolmogorov (FPK) diffusion equation. BBMV can fit Brownian motion (BM), Ornstein-Uhlenbeck (OU), and more complex evolutionary models. BM describes trait evolution as a random walk determined by a species mean trait value and the evolutionary rate parameter *σ*^2^. Under BM, trait values will drift randomly in proportion to the evolutionary time that separates them. The OU model additionally includes a long term mean trait value *θ* that species are attracted towards. The attraction is proportional to the *α* parameter, which is sometimes called the “rubber band” parameter (e.g., [113]) because it pulls drifting trait values back to *θ.* Using the FPK equation, BBMV extends OU models by allowing more than one long-term mean value and complex variation in the attractiveness of these mean values. BBMV models the probability density of a trait (*x*, GSA in our case) as a combination of an evolutionary rate *σ*^2^ and a constant deterministic force, proportional to the evolutionary potential function *V*(*x*). Species are attracted to minima of *V*(*x*) defined by *e*^-V(*x*)^. To allow a more intuitive interpretation, *e*^-V(*x*)^ is scaled using a normalization factor *N*, converting it into a macroevolutionary landscape *Ne*^-V(*x*)^. In this landscape, peaks correspond to trait values species are attracted towards, and peak height indicates how fast species move toward the trait value (i.e., how attractive the peak is). The shape of *V*(*x*) can be adjusted to compare models specifying different types of evolutionary landscapes. We used the polynomial equation suggested by [60, 61], *ax*^4^ + *bx*^2^ + *cx*, for *V*(*x*), bounded GSA (0,1), and fit all models with 200 discretization windows (Npts = 200). We then compared the AIC weights of the full model with up to two peaks (estimating *a*, *b*, and *c*), the OU model with up to one peak (*a* = 0), and the BM model without peaks (*a* = 0, *b* = 0, *c* = 0). We incorporated uncertainty in trait estimation by providing all individual GSA estimates to the lnL_BBMV function and determined the 95% confidence interval of the polynomial terms using the “Uncertainty_FPK” function. Because the full model only has two peaks when *b* is negative, we tested if the 95% confidence interval of *b* remains below 0.

### General phylogenetic regression approach

We tested a number of predictors of GSA evolution (see next section) using phylogenetic generalized least squares (PGLS) regression, using the *gls* function in the R package *nlme* (version 3.1). We incorporated phylogenetic signal in the residuals of the regression and simultaneously accounted for varying sample size, using the number of estimates for the dependent variable as weights (“~1/*N*_specimens_”). We fit evolutionary models (BM, *λ*, and OU) for the covariance in the residuals and selected the model with the lowest corrected AIC. Occasionally, both the *λ* and OU models had the same corrected AIC value, in which case we used the *λ* model. Due to this rule, the *λ* model was preferred in all analyses. Therefore, the PGLS covariances are best captured by a model that reduces the weight of the phylogeny compared to the expectation under BM. We checked the distribution of the phylogeny-corrected residuals for normality and profiled the likelihood of the parameter of the correlation structure (i.e., *λ*). In some smaller subsets of the data, we had issues with model convergence because the likelihood profile indicated λ<0. In these cases, we restricted *λ* to zero, making the model equivalent to a weighted ordinary least squares (OLS) regression. Since *R*^2^ values are problematic for OLS models [114], we calculated *R*^2^_pred_ using the R package rr2 [115] to show model fits. Specifically, we compared the model fit of an intercept-only model (*Y* ~ 1 + ε) with a model including the predictor(s) and the phylogeny (*Y* ~ *X*_i_ + *X*_j_ + ε | Ψ). *R*^2^_pred_ thus represents the predictive power of both the data and the phylogeny.

### Predictors of sex allocation

We performed univariate analysis, using four binary categorical predictors, namely the inferred mating syndrome (hypodermic vs. reciprocal, excluding the two species classified as intermediate), the received sperm location (at least some hypodermic sperm vs. sperm in antrum only), the sperm bristle state (bristles absent/reduced vs. bristles present), and the antrum state (thin vs. thickened), classified as in [43]. Next, we performed univariate PGLS with the estimate of per-site heterozygosity of the species for which we have a transcriptome (see above). Specifically, we log_10_-transformed the heterozygosity estimate and tested whether hypodermic insemination is associated with a reduction in heterozygosity, using a PGLS with the inferred mating syndrome as the predictor. We also used a PGLS analysis to evaluate the relationship between heterozygosity and GSA, first across all species, as well as restricted to the species showing the hypodermic or reciprocal mating syndrome, respectively.

Since GSA could change due to changes in testis size, ovary size, or both, we performed univariate PGLS analyses with testis size and ovary size as the dependent variables. First, we fit a model only including body size as the predictor to investigate the evolutionary allometry of each gonad (allometry models), performing a *t* test against a null model of an isometric slope of one. Second, we fit models that additionally included the inferred mating syndrome as a predictor to explore what changes in gonad size underlie differences in GSA (covariate models). Furthermore, we performed univariate PGLS analyses assessing whether GSA was predicted by body size. Additionally, to test the fundamental assumption of a sex allocation trade-off, we also performed a univariate PGLS of residual testis size on residual ovary size to determine if GSA changes can be seen as reallocation between sex functions.

Next, we explored the relationships between GSA and four reproductive morphology traits that may serve as indicators of the intensity of postcopulatory sexual selection (“morphological indicators”). These morphological indicators may also represent non-gonadal components of sex allocation in *Macrostomum*, with one trait summarising investment into the female genitalia (antrum complexity) and three traits representing aspects of male allocation (stylet length, sperm length, sperm bristle length). Specifically, this was done by performing univariate PGLS analyses with GSA as the dependent variable. Finally, we explored complex effects of specific trait combinations using multivariate PGLS analysis for GSA, using these four morphological indicators as well as body size as predictors. We performed all analyses across the entire dataset, as well as restricted to species classified as belonging to the hypodermic or reciprocal mating syndrome. Even though hypodermic species have low variability in antrum complexity and bristle length, we still performed PGLS analyses for these traits for consistency between the analyses. We do not correct for the resulting multiple testing since the data used varies slightly between tests. But we note that the *p* values were usually low and would, in most cases, remain significant after adjustment.

## Supplementary Information

**Additional file 1: Table S1.** Details on all specimens included in this study. **Table S2.** Mean species values for all variables. **Table S3.** Sample size per species for all quantitative traits. **Table S4.** PGLS analysis of gonadal sex allocation and various indicators of the inferred mating syndrome. **Table S5.** Heterozygosity estimates. **Table S6.** Univariate PGLS with GSA as the dependent variable. **Table S7.** Results from PGLS regression analysis of (A) testis size and ovary size on body size and (B) a covariate model including body size and the inferred mating syndrome. **Table S8.** PGLS of the residual testis and residual ovary size. **Table S9.** Multivariate PGLS of gonadal sex allocation using four sexual traits and body size as predictors.

**Additional file 2: Figure S1.** Gonadal sex allocation of species dependent on (A) received sperm location, (B) sperm bristle state, (C) antrum state, and (D) inferred mating syndrome. For the analysis in panel (D), the species belonging to the large clade only containing hypodermically mating species (clade I in Fig. 2) were removed to evaluate the robustness of the association shown in Fig. 3B. Boxes show the second and third quartile and the whiskers extend up to 1.5 times the interquartile range. Within each panel the main results of the PGLS analysis comparing the yellow and green data points (excluding the grey data) are given. Detailed results are in Additional file 1: Table S4.

**Additional file 3: Figure S2**. Per-site heterozygosity for all evaluated specimens. Specimens are grouped by species and ordered by mean heterozygosity. Colours indicate specimens collected from different sampling locations, likely corresponding to different populations. Heterozygosity estimates resulting from mapping to a common reference are indicated as squares. Variation within species is largely driven by differences between populations in species with intermediate values, while agreement between populations is high for low values.

## Data Availability

All data generated or analyzed during this study are included in this published article and its supplementary information files or in these Zenodo repositories: 10.5281/zenodo.2602479, 10.5281/zenodo.4543289, 10.5281/zenodo.5656981.

## Abbreviations

BM: Brownian motion
BBMV: Bounded Brownian motion with evolutionary potential
FPK: Fokker-Planck-Kolmogorov
GSA: Gonadal sex allocation
HI: Hypodermic insemination
ICC: Intraclass correlation coefficient
LSC: Local sperm competition
LMC: Local mate competition
OLS: Ordinary least squares
OU: Ornstein-Uhlenbeck
PGLS: Phylogenetic generalized least squares
