# Supplementary figures and images for: Mating behavior and reproductive morphology predict macroevolution of sex allocation in hermaphroditic flatworms
Source: BMC Biol. 2022 Feb 7;20:35. doi: 10.1186/s12915-022-01234-1 (PMC8822660; doi:10.1186/s12915-022-01234-1)

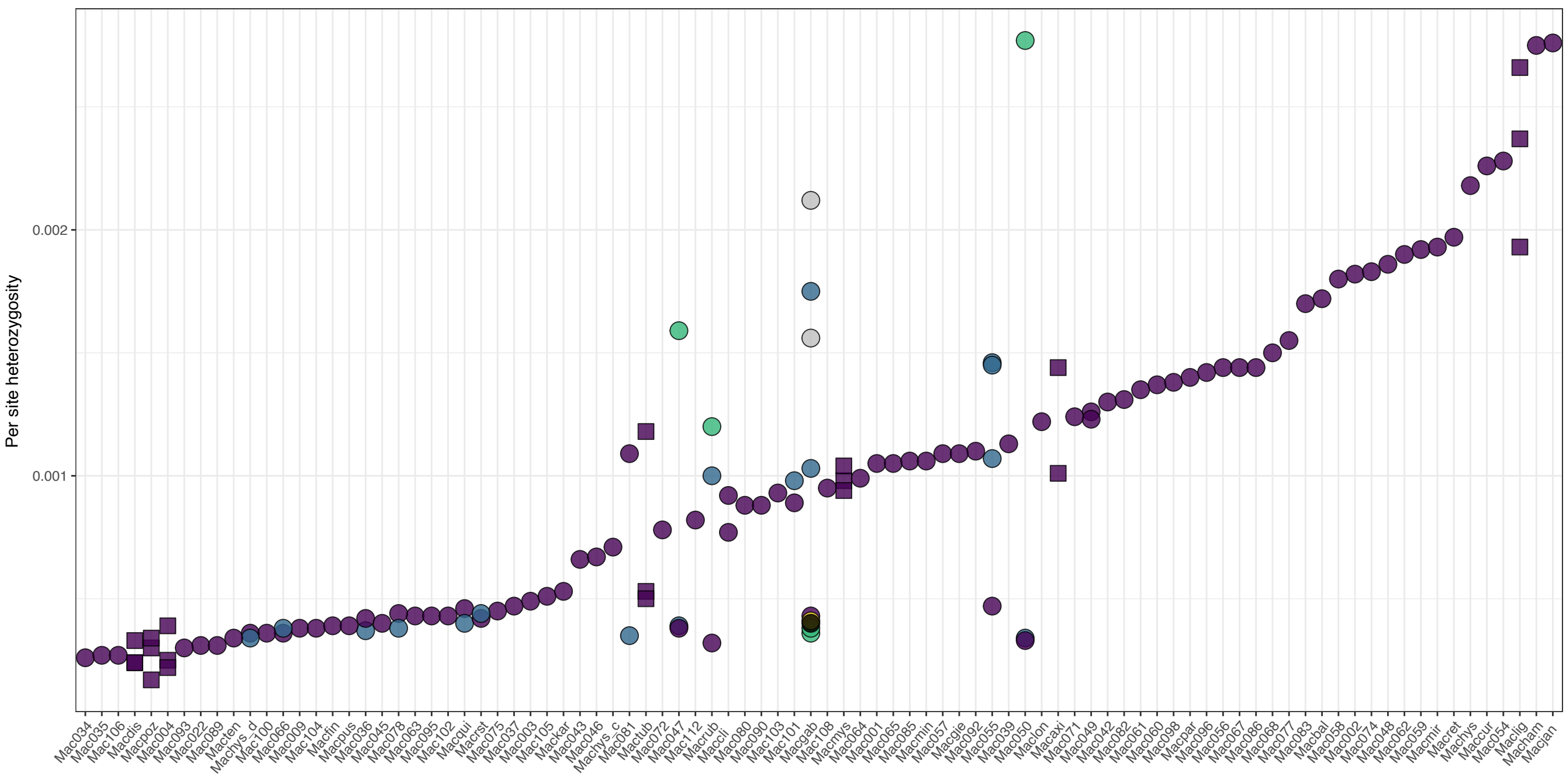

Supplement: Supplementary file 3 — Additional file 3: Figure S2. Per-site heterozygosity for all evaluated specimens. Specimens are grouped by species and ordered by mean heterozygosity. Colours indicate specimens collected from different sampling locations, likely corresponding to different populations. Heterozygosity estimates resulting from mapping to a common reference are indicated as squares. Variation within species is largely driven by differences between populations in species with intermediate values, while agreement between populations is high for low values. [file 12915_2022_1234_MOESM3_ESM.pdf]
