# Supplementary material for: Mating behavior and reproductive morphology predict macroevolution of sex allocation in hermaphroditic flatworms
Source: BMC Biol. 2022 Feb 7;20:35. doi: 10.1186/s12915-022-01234-1 (PMC8822660; doi:10.1186/s12915-022-01234-1)
Supplement: Supplementary file 2 — Additional file 2: Figure S1. Gonadal sex allocation of species dependent on (A) received sperm location, (B) sperm bristle state, (C) antrum state, and (D) inferred mating syndrome. For the analysis in panel (D), the species belonging to the large clade only containing hypodermically mating species (clade I in Fig. 2) were removed to evaluate the robustness of the association shown in Fig. 3B. Boxes show the second and third quartile and the whiskers extend up to 1.5 times the interquartile range. Within each panel the main results of the PGLS analysis comparing the yellow and green data points (excluding the grey data) are given. Detailed results are in Additional file 1: Table S4. [file 12915_2022_1234_MOESM2_ESM.pdf]

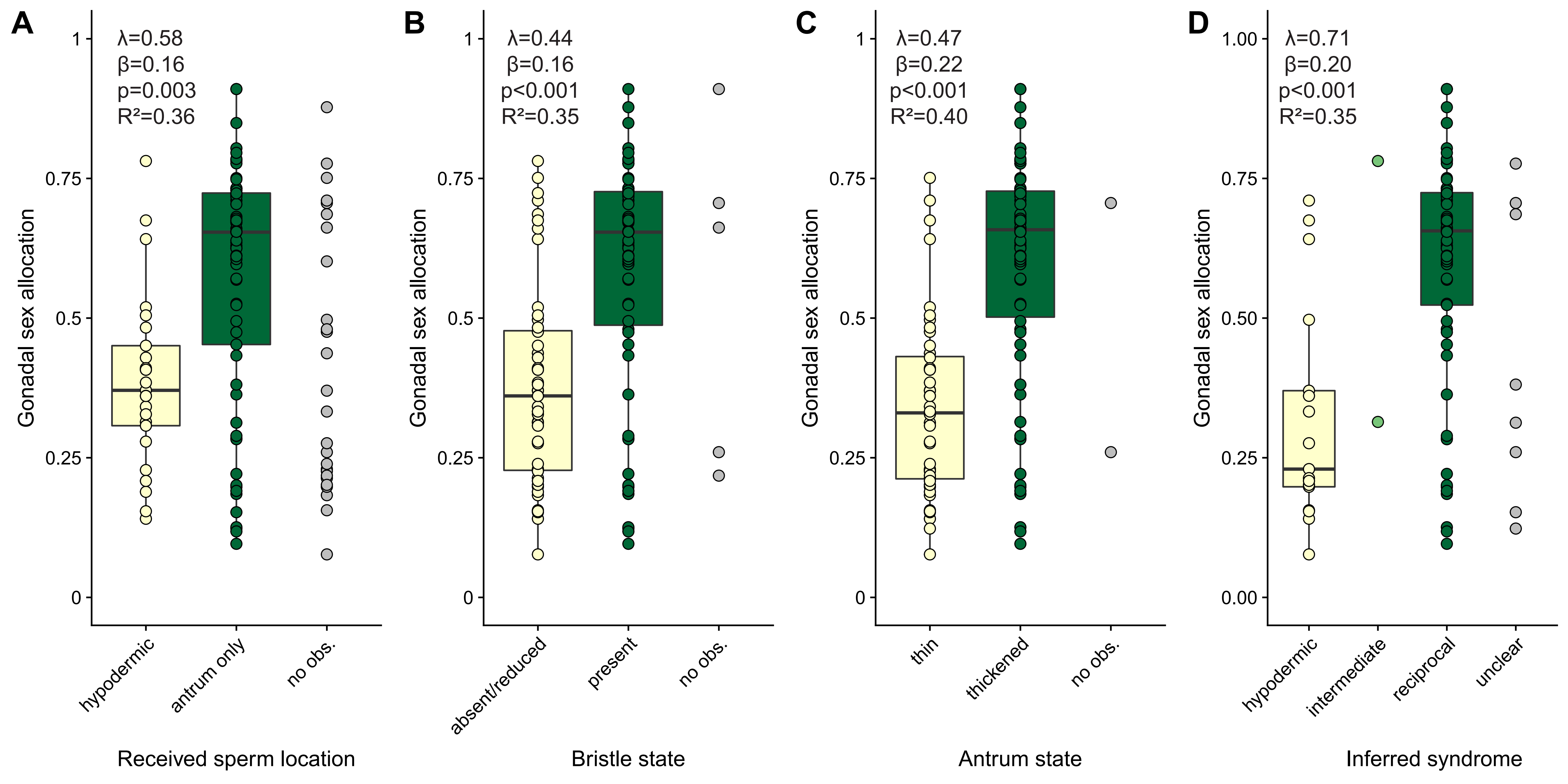
A
λ=0.58
β=0.16
p=0.003
R²=0.36
Gonadal sex allocation
hypodermic
antrum only
no obs.
Received sperm location
B
λ=0.44
β=0.16
p<0.001
R²=0.35
absent/reduced
present
no obs.
Bristle state
C
λ=0.47
β=0.22
p<0.001
R²=0.40
thin
thickened
no obs.
Antrum state
D
λ=0.71
β=0.20
p<0.001
R²=0.35
hypodermic
intermediate
reciprocal
unclear
Inferred syndrome
